# Differences Between Exergaming Rehabilitation and Conventional Physiotherapy on Quality of Life in Parkinson's Disease: A Systematic Review and Meta-Analysis

**DOI:** 10.3389/fneur.2021.683385

**Published:** 2021-08-09

**Authors:** Papamichael Elena, Solou Demetris, Michailidou Christina, Papamichail Marios

**Affiliations:** ^1^Department of Life and Health Sciences, School of Sciences and Engineering, University of Nicosia, Nicosia, Cyprus; ^2^Faculty of Medicine, University of Freiburg, Freiburg im Breisgau, Germany; ^3^PricewaterhouseCoopers Company Central Cyprus, Nicosia, Cyprus

**Keywords:** Parkinson's disease, conventional physiotherapy, quality of life, functionality, exergaming

## Abstract

Parkinson's disease (PD) is a neurodegenerative condition with both motor and non-motor symptoms affecting the quality of life (QoL) of older adults. Exergaming rehabilitation allows the interaction of the subject with digital games through the implementation of repetitive functional activities. Conventional physiotherapy uses patient-centered programs that include a variety of active exercises. The aim of this review was to look into the effectiveness of exergaming rehabilitation on the QoL of people with PD and compare it with conventional physiotherapy. Five electronic databases were searched for eligible studies until February 2021. For the statistical analysis, the mean, standard deviation, and 95% confidence interval were used to calculate effect sizes between groups. To determine heterogeneity, statistical index *I*^2^ was used. A total of 548 participants were included in 14 studies. Exergaming rehabilitation related with improved QoL (*p* = 0.687, 95% CI: −1.682 to −0.734), balance (*p* = 0.039, 95% CI: 0.364–13.689), (*p* = 0.018, 95% CI: 0.446–4.830), and gait (*p* = 0.005, 95% CI: 0.351–1.924). No significant difference was found between groups regarding the Unified Parkinson's Disease Rating Scale (*p* = 0.196, 95% CI: −5.970 to 1.225) and for the Timed Up and Go Test (*p* = 0.12, 95% CI: 0.446–4.830). Exergames as a rehabilitation method can be used to provide alternative interactive intervention with positive results for QoL in people with PD. Further investigation is needed to assess the effect on mental health in this population group.

## Introduction

Parkinson's disease (PD) is a progressive neurodegenerative disease that affects older people after the sixth decade of life. It involves motor and non-motor signs and symptoms (1, 2) and it is characterized by degeneration and progressive loss of dopamine neurons in the pars compacta of the substantia nigra (SNc), leading to disorganization, and dysfunction of the basal ganglia (3).

PD is the second most common neurodegenerative disease after Alzheimer's disease (4), affecting 1% of people older than 60 years of age (5). Countries with high industrial development, like the European countries and the USA, show high percentages of the disease in comparison with lower industrial development countries (6, 7). Until 2016, around 6.1 million cases of PD had been recorded worldwide, with 3.2 million showing disability problems and around 211,296 deaths recorded in 2016 (8).

Due to PD being a progressive disorder, treatment can be ongoing, adding to the cost that also depends on the severity of the condition and the needs of each patient. The total direct and indirect cost in Europe is around €14 billion per year (4), while in the USA, it is around $25.4 billion (9). It becomes obvious that this condition is an economic challenge for health services.

The main symptoms of the disease include cardinal signs that involve a number of complex motor signs (10) including resting tremor (4–6 Hz), rigidity, bradykinesia/akinesia, and loss of postural reflexes (5, 10). The existence of the cardinal signs limits function and activities of daily living (ADL), leading to a reduction in quality of life (QoL) (1). ADL limitations reduce social interaction function and independence (11, 12). Furthermore, a number of psychomotor, cognitive, and sensorial symptoms like pain, hyposmia, reduction of proprioception and kinesthesia, and decrease in memory and concentration have been reported (2). A large percentage of patients (40–50%) show emotional changes including anxiety disorders and depression, which lead to themselves noticing the symptoms and recognizing the disorder (13).

Physiotherapy can use a variety of interventions to treat psychomotor symptoms in PD based on the needs and goals set for each patient (4). Conventional physiotherapy (CPT) is one of the most common healthcare management methods used in PD (14) that provides a specified program of active exercises that use changes in the center of gravity (CG) and balance aerobic exercises (14, 15).

Exergaming rehabilitation (ER) is a broad spectrum that includes all the types of therapeutic immersion to project interactive digital exercises (16). As a rehabilitation method, it provides digital games and the user does exercises in order to achieve the game's outcomes (17). Adams et al. (17) defined ER as “videogames that use exertion-based interfaces to promote physical activity, fitness, and gross motor skills development”. ER is available with every equipment that projects digital exercise programs including non-immersive consoles, semi-immersive hybrid systems, and immersive virtual reality (VR) tools. In order for ER to function, the use of platforms, pads, video-consoles, and, most recently, VR headset and controller is essential (18).

The combination of ER with the use of a treadmill by patients with PD has shown positive results on gait, as stride length and balance were increased (19–21) and improvement in upper limb movement in particular oscillation of the arms (20). Finally, rehabilitation with the use of ER is found to significantly improve mental health in people with PD (21–23).

As patients with this specific health condition deteriorate with time, the constant burden on the psychomotor level can be unavoidable and can increase treatment cost (24). Exergaming methods have been used for rehabilitation purposes in recent years (25). This systematic review aims to identify, meta-analyze, and present the outcomes on ADL, physical and cognitive function, and QoL when using ER in the rehabilitation of people with PD. A comparison between ER and CPT results is a main goal of this review. Results are expected to aid understanding of the value to use ER, which will help clinicians and researchers in their decision-making.

## Methods

### Search Strategy

**Figure 1:**
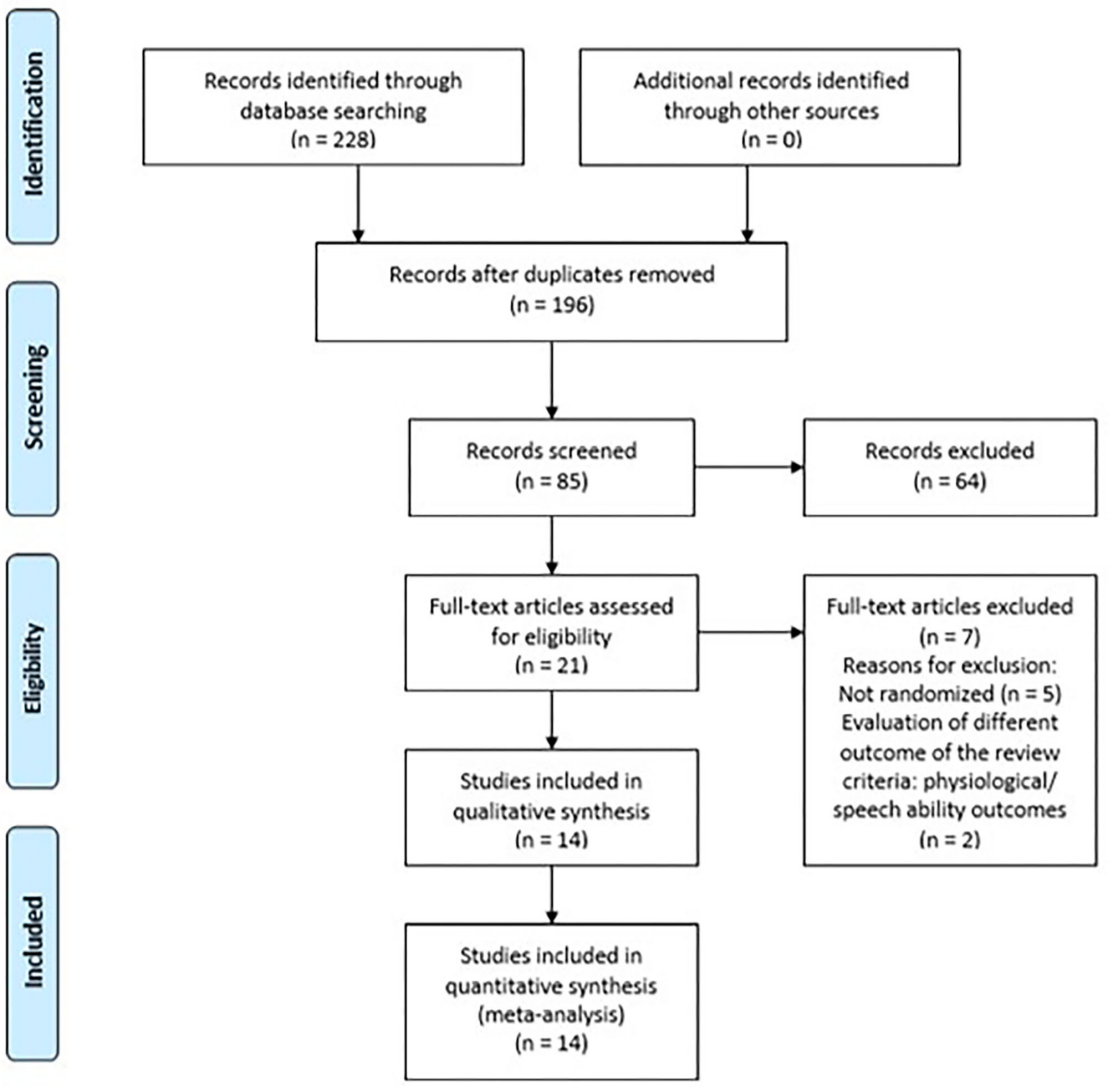
Preferred Reporting Items for Systematic reviews and Meta-Analyses (PRISMA) flowchart for the selection of the included studies.

This systematic review is registered with the PROSPERO database (CRD42020196946). The Preferred Reporting Items for Systematic reviews and Meta-Analyses (PRISMA) statement principles, using the population, intervention, control, and outcomes (PICO) model, have been followed (guidelines 2020). The following electronic databases, with no timeline or language restrictions, were searched: Medical Literature Analysis κ*αι* Retrieval System Online (MEDLINE/PUBMED), Physiotherapy Evidence Database (PEDro), Cochrane Controlled Trials Register (CENTRAL/CCRT), and Scientific Electronic Library Online (SciELO). Figure 1 presents details of the search procedure followed.

### Inclusion Criteria

The inclusion criteria were as follows: (1) randomized controlled trials (RCTs); (2) diagnosis of PD; (3) the experimental intervention to have used ER that included exergaming tools [This should involve video-consoles (Nintendo, X-box, etc.) for non-immersive tools, 3D programs with computers and cockpits for semi-immersive tools, and VR environment with headset for fully immersive tools.]; (4) the control group to have practiced CPT, which included any type of active exercise; (5) the study assessed QoL, physical function, and cognition.

### Quality Assessment of Studies

Data were extracted by one reviewer (PE) and revised by a second independent reviewer (SD). The studies that met the inclusion criteria were transferred onto the CADIMA system, which is an electronic tool that facilitates documentation in systematic reviews (26). The two reviewers (PE, SD) separately evaluated the studies, in two different timelines, June and August 2020, and then re-searched the literature for new studies in December 2020 and February 2021. The studies were evaluated in two phases. Phase 1 was conducted by reviewer PE, who screened the titles and abstracts for eligibility. Phase 2 was completed by reviewer SD who reviewed the full text of the previously selected studies. For assessing study bias, the PEDro Scale was used by both reviewers. The PEDro assessment tool was developed to evaluate methodological quality of clinical trials (27). No discrepancies were found during the study quality assessment, and results are presented in Table 1.

**Table 1:** PEDro assessment for the included RCTs.

**Study identification number**	**Eligibility criteria**	**Randomly allocated to groups**	**Concealed allocation**	**Blind subjects**	**Blind therapists**	**Blind assessors**	**Adequate follow-up**	**Intention-to-treat analysis**	**Between-group comparisons**	**Point estimates and variability provided**	**Total score**
Pompeu et al. (28)	Yes	Yes	No	No	No	Yes	Yes	Yes	Yes	Yes	7
Pedreira et al. (29)	Yes	Yes	Yes	No	No	Yes	No	No	No	Yes	5
Pazzaglia et al. (30)	Yes	Yes	No	Yes	No	No	No	Yes	Yes	Yes	6
Liao et al. (31)	Yes	Yes	Yes	Yes	No	Yes	Yes	No	Yes	Yes	8
Gandolfi et al. (32)	Yes	Yes	Yes	No	No	Yes	Yes	No	Yes	Yes	7
Fontoura et al. (33)	Yes	Yes	No	Yes	Yes	No	No	Yes	Yes	Yes	7
Feng et al. (34)	Yes	Yes	No	Yes	No	Yes	No	Yes	Yes	No	6
Allen et al. (35)	Yes	Yes	Yes	No	No	Yes	Yes	No	Yes	Yes	7
Pavez-adasme et al. (36)	Yes	Yes	No	No	No	No	No	Yes	Yes	Yes	5
Santos et al. (37)	Yes	Yes	Yes	No	No	Yes	Yes	No	Yes	Yes	7
Shen and Mak (38)	Yes	Yes	Yes	Yes	No	Yes	Yes	No	Yes	Yes	8
Shih et al. (39)	Yes	Yes	Yes	Yes	No	No	No	No	Yes	Yes	6
Yang et al. (40)	Yes	Yes	Yes	No	No	Yes	Yes	Yes	Yes	Yes	8
Tollar et al. (41)	Yes	Yes	No	Yes	No	Yes	No	Yes	Yes	Yes	7

*PEDro, Physiotherapy Evidence Database; RCT, randomized controlled trial*.

### Data Synthesis and Analysis

For the analysis, the statistical software SPSS 25.0 was used. Analysis was based on the mean, standard deviation (SD), and confidence interval (CI) for the evaluation of the effect sizes between groups. Statistically significant difference was set at <0.05 (42).

For the examination of homogeneity, Levene's test was applied. In order to have homogeneity, groups had to be equal, which means that homogeneity Sig index, or *p*-value, was set at >0.05. All types of immersion for exergames and VR programs were grouped together in the meta-analysis and compared against the control group.

A random-effects meta-analysis was performed with the use of the OpenMeta-analyst software (37). More specifically, the continuous random-effects DerSimonian and Laird model with 95% CI was used. To determine heterogeneity, statistical index *I*^2^ for the description of the variation between the studies was used. Significant level of heterogeneity of the index *I*^2^ was set at >75%. The weight assigned to each study was based on the variance and *t*^2^ value of each study. Furthermore, forest plots were used to illustrate the mean difference and CI between the experimental group and control group for each of the included studies. Multiple meta-analyses were performed in order to evaluate scales and outcomes used.

## Results

### Study Selection

The initial literature search detected 228 studies, but following screening of the title and abstract, only 21 remained for further examination. Following reading of the full text, seven studies were excluded. In particular, five studies were pilots of a clinical trial and two did not match the inclusion criteria for this review. One of the studies evaluated physiological variables and the other visuospatial and speech ability variables. Following the exclusion of these studies, 14 studies met the inclusion criteria of this systematic review (28–41).

Based on the PEDro Scale, an average score of 6.7/10 for the included studies was found. The total score of the scale ranged from 0 to 10, with scores of 9–10 considered “excellent,” 6–8 “good,” 4–5 “fair,” and 0–3 “poor” (43). In this systematic review, as shown in Table 1, only two studies were given a “fair” score (29, 36), while the rest of the studies received a “good” quality score (28, 30, 32–35, 37, 39, 41). The highest score was 8/10, and it was given to three studies (31, 38, 40).

The Levene's test, done to examine the clinical characteristics of the studies, found homogeneity (*p* > 0.05) between the studies, which allowed the meta-analysis to be performed. Six different group analyses had a heterogeneity score of *p* > 0.05 and thus were further meta-analyzed for QoL, ADL, and physical function. Cognitive function did not pass the set value for heterogeneity (*p* = 0.039) and was thus not included in any further analysis. **Table 4** presents the results of the meta-analysis of the outcomes that passed heterogeneity examination.

### Participant Characteristics

In total, 548 people with PD were included in this review from 14 different studies (248 in the experimental group, 249 in the control group, and 51 in a different third group). A total of 59.9% (328 patients) were males, and 37.8% (207 patients) were females. In the study of Pedreira et al. (29), the gender for 13 participants was not reported. The mean age of the target group was 67.3 years (±2.877), while the mean grade of the severity of the disease, as evaluated by the Hoehn and Yahr scale (44), ranged between 1 and 3. The mean duration of the disease in years was 6.75 (±1.488), as summarized in Table 2.

**Table 2:** Demographic characteristics of the participants.

**Study**	**Country**	**Gender**	**Age (SD)**	**Disease Duration**	**Disease Classification**
Pazzaglia et al. (30)	Italy	Female = 16	71 (8.5)	6 (± 6.29)	UPDRS III 24
		Male = 35			
Pavez-adasme et al. (36)	Chile	Female = 3	66.6 (8.1)	4.5 (± 2.6)	H&Y 1–3
		Male = 5			
Tollar et al. (41)	The Netherlands	Female = 38	69.3 (4.35)	7.4 (± 2.04)	H&Y 2–3
		Male = 36			
Santos et al. (37)	Brazil	Female = 14	64.2 (8.5)	7.1 (± 0.5)	H&Y 1–3
		Male = 31			
Feng et al. (34)	China	Female = 12	67.1 (4.71)	6.8 (± 1.44)	H&Y 2–4
		Male = 16			
Allen et al. (35)	Australia	Female = 15	67.9 (7.9)	5.6 (± 5)	UPDRS III 40
		Male = 23			
Fontoura et al. (33)	Brazil	Female = 4	63 (7)	n/g	H&Y 1–3
		Male = 16			
Gandolfi et al. (32)	Italy	Female = 25	68.6 (8.2)	6.8 (± 3.85)	H&Y 2.5–3
		Male = 51			
Yang et al. (40)	Taiwan	Female = 9	75.2 (7.35)	10 (± 3.85)	H&Y 2–3
		Male = 14			
Shih et al. (39)	Taiwan	Female = 4	68.1 (9.81)	4.6 (± 4.29)	H&Y 1–2
		Male = 18			
Liao et al. (31)	Taiwan	Female = 19	65.6 (7.46)	7 (± 2.83)	H&Y 1–3
		Male = 17			
Shen and Mak (38)	China	Female = 24	64.3 (8.25)	7.3 (± 4.15)	H&Y 1–3
		Male = 27			
Pedreira et al. (29)	Brazil	Female = 9	63.65 (8.25)	7.9 (± 5.6)	H&Y 1–2.5
		Male = 22			
Pompeu et al. (28)	Brazil	Female = 15	67.4 (8.1)	n/g	H&Y 1–2
		Male = 17			

### Interventions

All the included studies used exergaming training as a rehabilitation intervention for the experimental group. Nine studies applied non-immersive equipment (28, 30–32, 34, 35, 38, 39, 41), while four studies used semi-immersive tools (29, 36, 37, 44). Only one study fully utilized immersive equipment (30). Details are summarized in Table 3. All studies used CPT for their control groups, with one of them (39) offering an additional one-off fall prevention education session. Two of the studies (37, 41) contained a third interventional group. In particular, in one study (39), the third group was told to continue with their ADL and they did not receive any physiotherapy intervention. In the other one (37), the third group received a combined ER and CPT interventional program. Details of all groups are presented in Table 3.

**Table 3:** Characteristics of the interventions.

**Study**	**Participants (*N*) Experimental/control group**	**Length of intervention in minutes**	**Frequency of intervention**	**Duration of intervention (weeks)**	**ER intervention**	**CPT intervention**	**Follow-up**
Pazzaglia et al. (30)	51 25/26	40	3	6	VR NIRVANA (function and coordination exercises)	Joint mobilization, respiratory balance and coordination exercises, gait	n/g
Pavez-adasme et al. (36)	8 4/4	45	2	6	Nintendo Wii Fit (Strength, balance, aerobic and stretching exercises)	Muscle strength, aerobic exercises, balance and stretching exercises	n/g
Tollar et al. (41)	74 25/25/24	60	5	5	Microsoft X-Box Kinect(motor control and stability exercises, balance)	CYC Group: Balance and aerobic exercises	n/g
						CG: Continuation of ADL	
Santos et al. (37)	45 15/15/15	50	2	8	Nintendo Wii Fit (games of boxing, soccer heading)	NWCE group: Combination of EG and CG training	n/g
						CG: PNF exercises, gait	
Feng et al. (34)	28 14/14	45	5	12	VR training (Balance, coordination and stretching exercises, gait)	Aerobic exercises, coordination, balance and stretching exercises, gait	n/g
Allen et al. (35)	38 19/19	n/a	3	12	VR Unity games (coordination and cognitive training)	General exercises and continuation of ADL	n/g
Fontoura et al. (33)	20 10/10	60	2	5	Microsoft X-Box Kinect (Functional, muscle strength, ROM and coordination exercises)	Stretching, muscle strength and balance exercises, gait	n/g
Gandolfi et al. (32)	76 38/38	50	3	7	TeleWii, Nintendo Wii, balance board (Stretching, balance and functional exercises)	Stretching and balance exercises	70
Yang et al. (40)	23 11/12	50	2	6	VR training balance board (Stretching, balance and functional exercises)	Balance and object manipulation exercises	20
Shih et al. (39)	22 11/11	50	2	8	Microsoft Kinect Sensory (Balance and coordination exercises, gait)	Balance, coordination, muscle strength exercises, gait	n/g
Liao et al. (31)	36 12/12/12	60	2	6	Wii Fit exergaming (yoga, balance and muscle strength exercises)	TE group: Muscle strength, balance and stretching exercises	35
						CG: Fall prevention education program	
Shen and Mak (38)	51 26/25	60	3	12	Computerized Dancing System, Smart-EquiTest Balance Master (Postural control and coordination exercises, gait, sit to stand and gait at home)	Lower limb muscle strength and physical condition exercises, gait at home	35
Pedreira et al. (29)	44 22/22	40	3	4	Nintendo Wii (boxing)	Balance and muscle strength exercises, gait	n/a
Pompeu et al. (28)	32 16/16	60	2	7	Wii Fit Exergaming (balance and cognitive training)	Balance, trunk rotations and transition of CB	32

*n, number of participants; ER, Exergaming rehabilitation; VR, virtual reality; ROM, range of motion; EG, experimental group; CG, control group; ADL, activities daily living; NWCE, Nintendo Wii Conventional exercise; TE, traditional exercise; CB, central body; n/g: not given*.

The characteristics of the intervention, including setting, frequency, and duration of the intervention, as well as number and age of participants, showed homogeneity (*p* = 0.98, 1.01, 0.58, 0.98, and 0.89, respectively). The majority of the studies (85.7%) took place in an outpatient setting.

The duration of interventions ranged between 40 and 60 min, while one study did not provide information about the duration of the intervention (35). Half of the studies practiced the rehabilitation program twice a week (28, 32, 33, 35–38), five studies three times per week (29, 31, 34, 39, 41), and two studies five times per week (30, 40). The total duration of the rehabilitation program ranged between 4 and 12 weeks, with the most common total duration being 6 weeks (28.6%).

### Quality of Life: Parkinson's Disease Questionnaire-39

**Figure 2:**
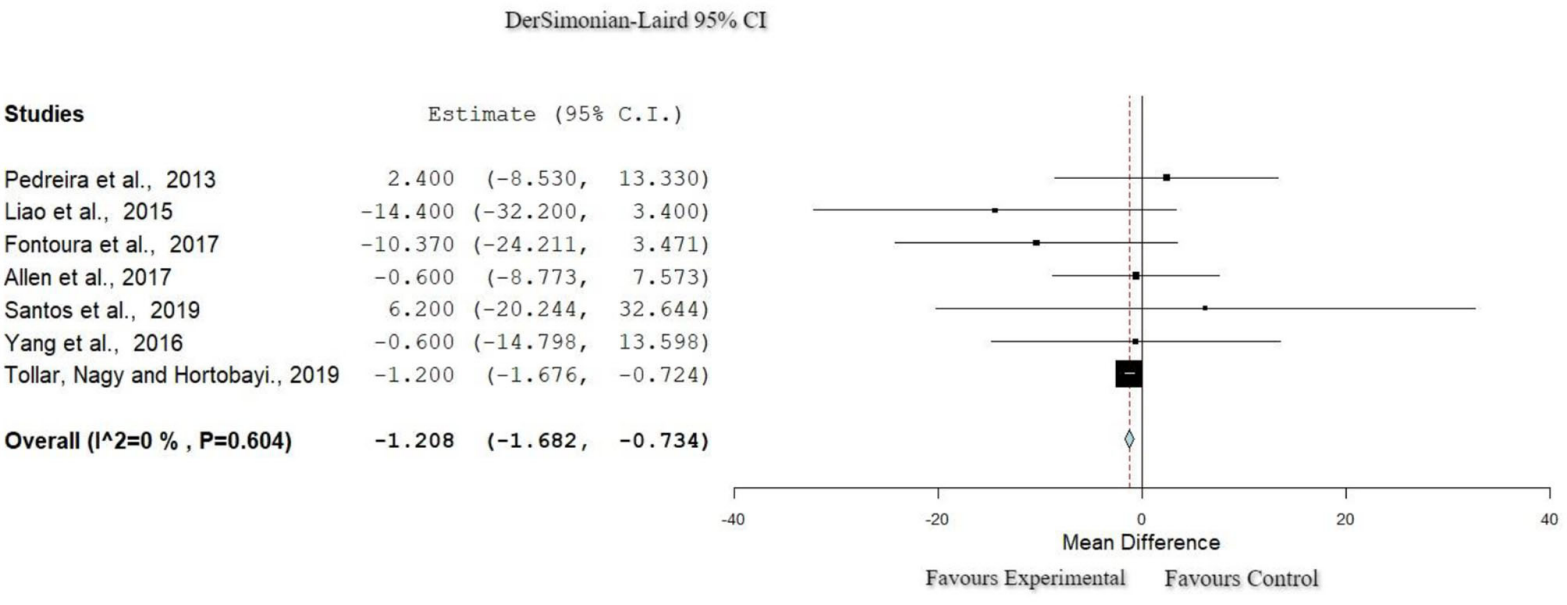
Forest plot for QoL: PDQ-39. The red dash line represents the weighted mean difference of the included studies. The blue figure represents the weighted 95% CI of the included studies. PDQ-39, Parkinson's Disease Rating Scale-39; QoL, quality of life.

Seven studies (28, 30, 33, 39, 41, 43, 44) were included in the meta-analysis of QoL (207 participants in total). All studies used the Parkinson's Disease Questionnaire-39 (PDQ-39), and all applied exergames for the experimental group. For the control group, all of them (30, 33, 41, 43, 44) offered active exercises as a rehabilitation program. To examine the effects of the interventions, the post-intervention data presented in these publications were meta-analyzed. As shown in Figure 2, this meta-analysis had zero percentage of heterogeneity (*I*^2^ = 0%, Het. *p* = 0.604), and a statistically significant difference in favor of the experimental group (*p* < 0.001, 95% CI: −1.682 to −0.734) for the QoL was found. Results are presented in Table 4.

**Table 4:** Results of the meta-analysis.

**Study**	**Weights**	**PDQ-39**
**QoL**		
Pedreira et al. (29)	0.18%	1.20 [1.682, −7.34]
Liao et al. (31)	0.07%	(0.242)
Fontoura et al. (33)	0.11%	*p* < 0.001
Allen et al. (35)	0.33%	
Santos et al. (37)	0.03%	
Yang et al. (40)	0.11%	
Tollar et al. (41)	99.14%	
**Study**	**Weights**	**ABC**
**ADL**		
Gandolfi et al. (32)	61.58%	7.02 [0.364, 13.68] (3.39) *p* = 0.039
Shen and Mak (38)	38.41%	
		**UPDRS II**
Pompeu et al. (28)	32.51%	−2.37 [−5.97, 1.225]
Fontoura et al. (33)	31.45%	(1.83)
Tollar et al. (41)	36.03%	*p* = 1.96
**Study**	**Weights**	**BBS**
**Function**		
Pompeu et al. (28)	19.06%	2.63 [0.446, 4.83]
Pazzaglia et al. (30)	10.08%	(1.11)
Feng et al. (34)	14.08%	*p* = 0.018
Santos et al. (37)	13.47%	
Shih et al. (39)	18.17%	
Yang et al. (40)	9.50%	
Tollar et al. (41)	15.60%	
		**TUG**
Liao et al. (31)	15.21%	−0.97 [−2.212, 0.258]
Feng et al. (34)	8.11%	(0.63)
Pavez-adasme et al. (36)	33.99%	*p* = 0.121
Santos et al. (37)	24.19%	
Shih et al. (39)	16.90%	
Yang et al. (40)	1.56%	
		**DGI**
Pazzaglia et al. (30)	8.58%	1.13 [0.351, 1.924]
Santos et al. (37)	9.66%	(0.40)
Tollar et al. (41)	81.75%	*p* = 0.005

*QoL, quality of life; ADL, activities of daily living; PDQ-39, Parkinson's Disease Rating Scale-39; ABC, Activities-Specific Balance Confidence Scale; UPDRS II, Unified Parkinson's Disease Rating Scale Part II; BBS, Berg Balance Scale; TUG, Timed Up and Go; DGI, Dynamic Gait Index*.

*Data are presented as standardized mean difference [95% CI], (standard error), p-value*.

### Activities of Daily Living: Activities-Specific Balance Confidence Scale

**Figure 3:**
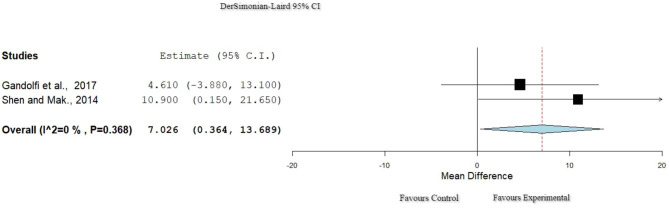
Forest plot for ADL: ABC. The red dash line represents the weighted mean difference of the included studies. The blue figure represents the weighted 95% CI of the included studies. ABC, Activities-Specific Balance Confidence Scale; ADL, activities of daily living.

Two studies (30, 35) used the Activities-Specific Balance Confidence Scale (ABC) to assess ADL. There was null heterogeneity (*I*^2^ = 0% Het. *p* = 0.368, Figure 3), and in total, 115 participants were extracted from these studies. The results (Table 4) showed a significant improvement for the ER group (*p* = 0.039, 95% CI: 0.364–13.689).

### Activities of Daily Living: Unified Parkinson's Disease Rating Scale II

**Figure 4:**
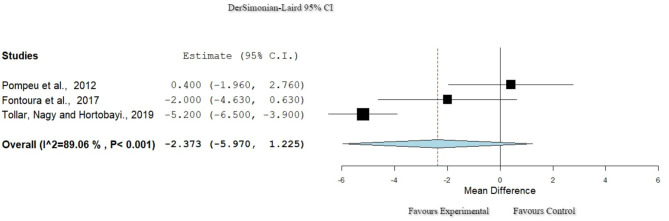
Forest plot for ADL: UPDRS II. The red dash line represents the weighted mean difference of the included studies. The blue figure represents the weighted 95% CI of the included studies. ADL, activities of daily living; UPDRS II, Unified Parkinson's Disease Rating Scale Part II.

Three studies (29, 32, 38) used the Unified Parkinson's Disease Rating Scale Part II (UPDRS II) to assess ADL. Heterogeneity, though high, passed the set criteria for inclusion in this meta-analysis (*I*^2^ = 89%, Het. *p* < 0.001; Figure 4). A total of 101 participants were included in these studies, and there was no significant difference between the groups (*p* = 0.196, 95% CI: −5.970 to 1.225).

### Function: Berg Balance Scale

**Figure 5:**
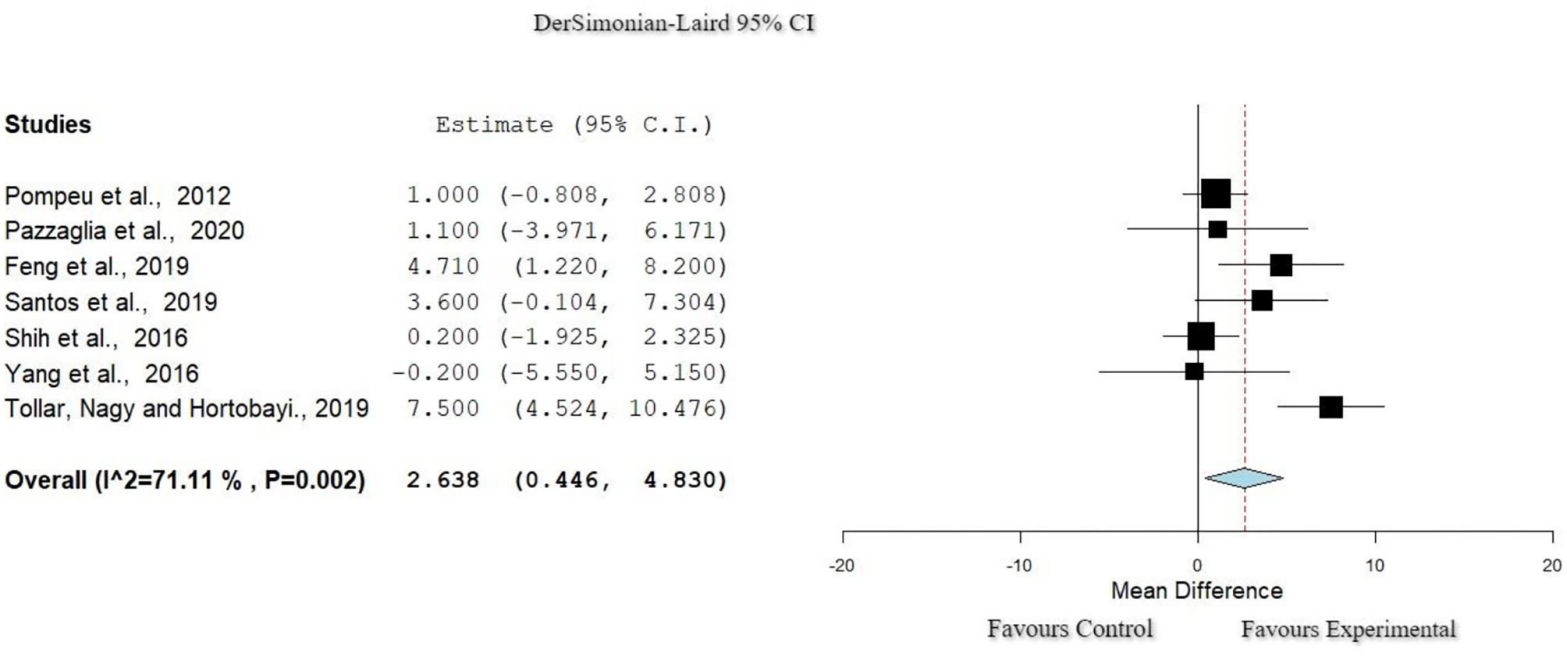
Forest plot for Function: BBS. The red dash line represents the weighted mean difference of the included studies. The blue figure represents the weighted 95% CI of the included studies. BBS, Berg Balance Scale.

Seven studies (29, 31, 33, 38, 40, 41, 43) used the Berg Balance Scale (BBS) to assess function. In the Figure 5 studies showed a relatively high heterogeneity (*I*_2_ = 71%, Het. *p* = 0.002). Based on the data extracted, a significant improvement was observed in the experimental group in comparison to the control group (*p* = 0.018, 95% CI: 0.446–4.830).

### Function: Timed Up and Go

**Figure 6:**
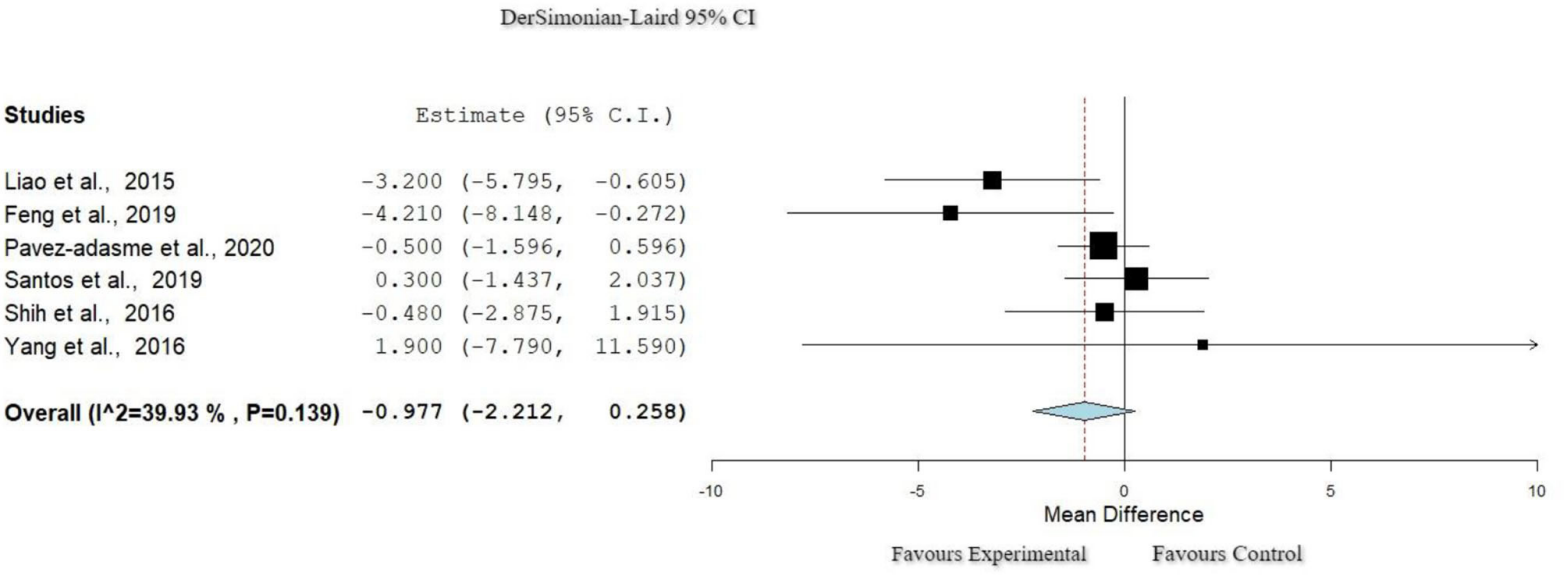
Forest plot for Function: TUG. The red dash line represents the weighted mean difference of the included studies. The blue figure represents the weighted 95% CI of the included studies. TUG, Timed Up and Go.

The Timed Up and Go (TUG) scale was used in six studies (31, 33, 37, 39, 41, 43). As seen in Figure 6, heterogeneity was relatively small (*I*^2^ = 39.9%, Het. *p* = 0.139). Meta-analysis did not indicate any significant difference in favor of any of the groups (*p* = 0.12, 95% CI: 0.446–4.830).

### Function: Dynamic Gait Index

**Figure 7:**
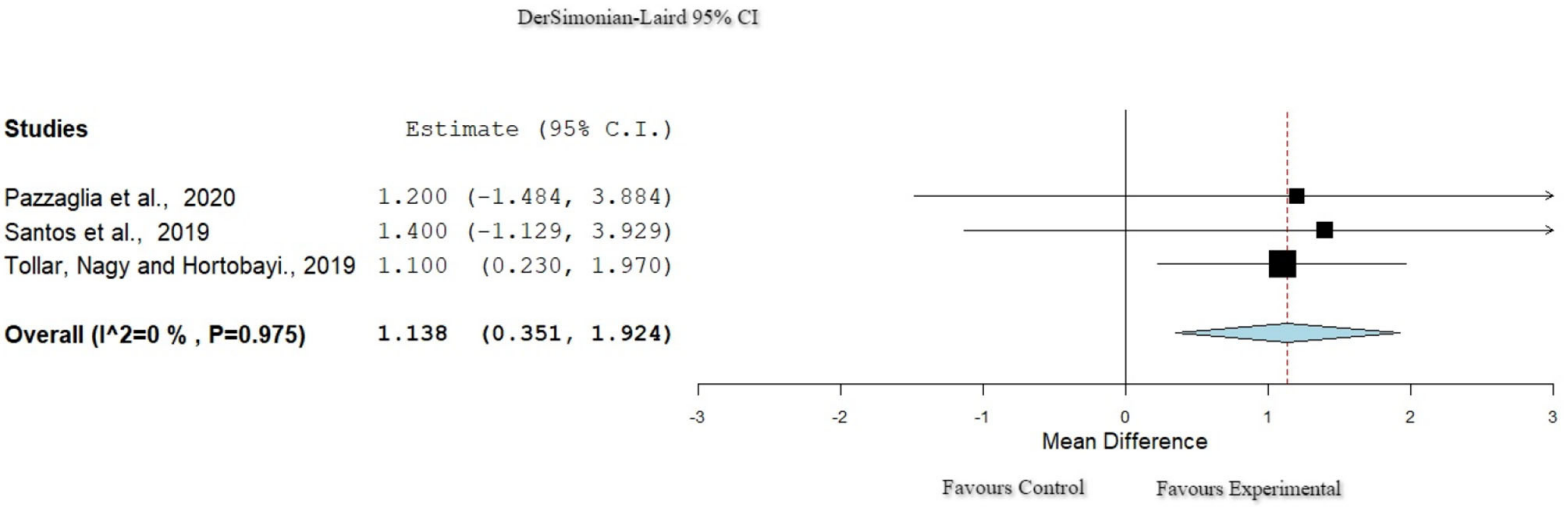
Forest plot for Function: DGI. The red dash line represents the weighted mean difference of the included studies. The blue figure represents the weighted 95% CI of the included studies. DGI, Dynamic Gait Index.

Three studies (38, 40, 43) used the Dynamic Gait Index (DGI) to evaluate function. The studies showed null percentage of heterogeneity (*I*^2^ = 0%, Het. *p* = 0.975) as presented in Figure 7. The results of the meta-analysis found a statistically significant improvement in function for the experimental group (*p* = 0.005, 95% CI: 0.351–1.924).

## Discussion

This review and meta-analysis aimed to evaluate the effects of ER on QoL, ADL, and physical and cognitive function in comparison with CPT in people with PD. Following a systematic examination of the major literature databases, 14 studies met the inclusion criteria and were meta-analyzed. All the included studies were published within the last 10 years, and they were RCTs using ER and CPT as their intervention rehabilitation methods. The data were checked for homogeneity, and several statistical tests were used to conduct the meta-analysis. The total pooled participant size was 548 people with PD.

The primary outcome evaluated in this systematic review was QoL, and secondary outcomes were ADL, cognitive function, and physical function. The identified studies used different evaluation scales to assess outcomes of their populations, and as such, several meta-analyses were conducted. To assess QoL, seven studies used the PDQ-39 scale (28, 30, 32, 33, 36, 38, 41), one study used the SF-36 (30), and one used the PDQ-8 scale (32). All studies used baseline and post-intervention evaluations. Follow-up evaluations were conducted in five studies (32, 34, 37, 38, 41). Most specifically, three studies had a 1-month follow-up (30, 37, 40), two studies had a 2-month follow-up (32, 38), and the longest follow-up duration was 12 months for one study (38).

The results of the meta-analysis showed that ER significantly improved QoL in comparison with CPT in people with PD (*p* < 0.001), which is in agreement with studies found in the literature (32, 33, 35, 40, 45). Other published systematic reviews that used VR methods (but did not compare with CPT) used the PDQ-8 scale to assess QoL and found significant improvements (32, 39, 41). In the current systematic review, only one study (35) used the PDQ-8, and they reported improvements in QoL as well. For the control group, only one study, which used the PDQ-39, showed a significant improvement in QoL (35).

The study of Pazzaglia et al. (30) used the SF-36 to assess QoL and was the only study not to find any significant difference between the experimental group and the control group. The mentioned assessment tool is a commonly used questionnaire for the evaluation of psychomotor variables. On the other hand, application of the PDQ-39 questionnaire seems to be more useful for the evaluation of the QoL, as it presents higher Cronbach's alpha index (0.76–0.93) regarding the correlation of the emotional changes of the people with PD with their status of health (46, 47). This concludes with the necessity of similar highly confident reliability use of the appropriate evaluation scales between clinical trials.

Three studies included a third intervention group using different approaches to improve QoL for the target group (28, 30, 32). The study of Santos et al. (37) combined ER with CPT for the intervention program of the third group, where the results showed no statistical difference for the effect size but an important magnitude of improvement of the combined group in comparison with the other groups. The combination of the two programs offers alternative solutions that cover the goals of the intervention in a multifactorial way. The reported study was the only RCT that applied this combined rehabilitation method, demonstrating the need for the evaluation of the effectiveness of the addition of ER to CPT for the QoL of the people with PD.

PD includes a large spectrum of symptoms that affect in a multifactorial way the QoL of this population group. The type of clinical setting can reveal different results to the wellbeing of the people with PD, since the outpatient setting follows an emotional approach for the mental improvement of the people (48). As mentioned by Gumber et al. (49), hospitalization of people presents an economic and emotional burden that affects their QoL. Furthermore, the outpatient setting resulted in a lower symptom burden on the population group and enhancement of the health-related quality of life (HRQoL) of the neurological patients and their families (50), confirming the findings of the studies that used outpatient setting in this systematic review. More specifically, 12 of the included studies (28–30, 32, 33, 35–41) took place in outpatient settings and only two in inpatient settings (31, 34). The two studies conducted in an inpatient setting showed improvement on the measured outcomes as the outpatient setting. More specifically, the study of Liao et al. (31) that used inpatient setting presented an increase of physical, emotional, and social function of the population group. In contrast to the findings of this systematic review, the study of Rajan et al. (51) showed positive effects on the reported QoL of the participants who received outpatient care in comparison with the standardized inpatient model setting (51). However, these results can be explained by the fact that the authors used different methodological approaches and they did not include studies with exergaming rehabilitation.

Physiotherapy can play a crucial role in the health management of people with PD (52). Multiple techniques can be applied in order to achieve goals aiming to improve motor skills and gait. Interventions that include exergaming methods seem to enhance both balance and QoL (53). These findings are supported by the current systematic review. The usage of ER intervention results in important improvements in function, as progress is found in balance and gait (48, 54). For the function, seven studies (28, 30, 34, 37, 39–41) used the BBS to measure the balance of the population. All the included studies showed a significant improvement of the balance through the application of ER comparing the pretest and posttest measurements. However, other studies found that function was not significantly more improved when using ER in comparison with when using CPT (29, 32, 34, 36, 39). In the study of Feng et al. (34), a significant improvement for the control group, in comparison with the CPT group, was found when using non-immersive VR. The application of immersive exergaming in a fully virtual environment showed a significant improvement for the experimental group with increase of function (34) in contrast to previous studies where non-immersive tools of ER programs were used (35, 40). Furthermore, three studies with high homogeneity used the DGI scale for the evaluation of the dynamic gait, presenting improvement of the mentioned outcome for the experimental group (30, 37, 41). The repetitive provision of sensory–motor stimulus with ER facilitates the interaction of the user with the environment, provoking enhancement of the functional outcomes like dynamic gait (30), as has been reported by the results of this systematic review.

The improvement in QoL and function when using exergaming programs leads to improvements in mental health by decreasing stress and depression (55, 56). People with PD have difficulties in executing ADL, which impact their QoL, leading to further emotional and physical limitations (57, 58). For the assessment of ADL, the most commonly used scale is UPDRS (51, 52), which is a valid scale to examine the relationship between disease severity and ADL (59). The usage of an appropriate evaluation questionnaire is considered to be of high importance in order for aspects that can limit the autonomy and functional ability of the target group to be identified (60).

The study of Hariz and Forsgren (61) indicated that changes to posture and balance limit motor capacity and communication skills, leading to a decrease in ADL capability. As found in this analysis, improvements in QoL and function related to improvements in ADL of the experimental groups (33, 35, 37, 38, 40, 41), while there was no such correlation with the improvement in the control group (29, 33, 38).

The small number of studies identified, in this systematic review, to have examined cognition used a variety of assessment scales not allowing a meta-analysis to be conducted. The effectiveness on cognitive function for people with PD is, thus, ambiguous, since only two studies showed improvement with ER and CPT (36, 39). The study of Pedreira et al. (29) did not show any significant improvement for cognition. The studies used different evaluation scales to measure outcomes, as two of them used the Montreal Cognitive Assessment (MoCA) scale (36, 39) and one used the PDQ-39 (29). The systematic review of Triegaardt et al. (54) mentioned improvement in the measurements of the MoCA scale for cognition, confirming the findings of this review on the cognitive function of people with PD, especially when using ER.

ER creates a safe environment that offers physical and cognitive training. The cognitive interaction can lead to cognitive improvements and better ADL skills (59). Active exercises in combination with visual and auditory stimuli offered by the ER increase skill repetitions and velocity and raise endurance (62). The kind of immersion created by VR differs based on the cost and the features of the equipment. Non-immersive applications have significantly lower cost compared to the fully immersive tools, which provide more realistic gamification. The non-immersive equipment and semi-immersive equipment are easier to apply as a means of therapy and can be used as home care intervention (61, 63). Even though this variable was not evaluated in this review, currently, the ER intervention especially with VR equipment is more expensive than CPT, but in the future, financial affordability is expected, which will enable easier access for the population overall and will facilitate its usage as a rehabilitation method (64, 65).

In summary, the studies included in this systematic review applied a variety of immersion tools for the experimental intervention. Most of the studies used non-immersion application (29, 30, 33–35, 38, 40–42, 62). A semi-immersion intervention tool, aiming to provide more realistic and accurate stimuli during training (40) or aiming to produce multiple tactile and auditory stimuli simultaneously (38), was also used. Only one study used full-immersion program as intervention (30), which limits conclusions to be made.

ER offers a wide range of options, based on users' needs, aiming to promote rehabilitation and offer psychomotor benefits (25). As an interventional tool, ER can be applied for people with disabilities, where the subjects experience functional activities within digital or virtual environment, without external constraints (66). Exergaming and virtual rehabilitation could facilitate tele-physiotherapy sessions and promotes access to rehabilitation (66). Exergaming ensures multiple and targeted repetitions and offers the opportunity to apply home based therapy (37, 64, 67). The combination of ER with CPT, can contribute to patients' education while promoting physical activity, improving psychomotor behaviors, an thus advances health (68–71).

The average PEDro score in this review was 6.7, which is considered to be good (43). Lack of double blinding and intention to treat in some of the studies is a methodological limitation and needs to be considered when designing future studies. Because of the small number of studies that compared the two different intervention techniques, a conclusion on the most efficient treatment cannot be made. The included studies presented numerous and interesting findings for the use of ER, although the different types of immersions that have been used did not provide a clear picture of the most adequate.

Multiple ER can be used to provide alternative interactive interventions. More future research is essential to further evaluate the benefits of this type of intervention in PD rehabilitation. The application of more advanced technological ER systems provides variability of gamification and simultaneous combination of exergaming programs that allow the execution of more realistic activities, raising the physical and emotional interaction between the individual and the environment. This gives the opportunity to evaluate the use and effectiveness of the intervention in mental health and motivation for people with PD, which should be considered in future studies.

In conclusion, the use of ER as an intervention tool can meet the needs and abilities of people with PD, as these systematic review and meta-analysis have found positive results in function and QoL. Only a small number of studies compared ER with CPT, and thus, future studies should follow such comparison designs. In addition, few studies examined the QoL in patients with PD and an even smaller number of studies compared it with the use of CPT. It is essential that more research is done in order to have more accurate data on this particular topic.

## Data Availability Statement

The original contributions presented in the study are included in the article/supplementary material, further inquiries can be directed to the corresponding author/s.

## Author Contributions

PE, SD, and MC contributed to conception and design of the study. SD and PE organized the database. PM and PE performed the statistical analysis. PE wrote the first draft of the manuscript. PE and MC wrote sections of the manuscript. MC reviewed the writing and organization of the study. All authors contributed to manuscript version, read, and approved the submitted version.

## Conflict of Interest

PM was employed by company PricewaterhouseCoopers. The remaining authors declare that the research was conducted in the absence of any commercial or financial relationships that could be construed as a potential conflict of interest.

## Publisher's Note

All claims expressed in this article are solely those of the authors and do not necessarily represent those of their affiliated organizations, or those of the publisher, the editors and the reviewers. Any product that may be evaluated in this article, or claim that may be made by its manufacturer, is not guaranteed or endorsed by the publisher.
